# Benchmarking germline CNV calling tools from exome sequencing data

**DOI:** 10.1038/s41598-021-93878-2

**Published:** 2021-07-13

**Authors:** Veronika Gordeeva, Elena Sharova, Konstantin Babalyan, Rinat Sultanov, Vadim M. Govorun, Georgij Arapidi

**Affiliations:** 1 Federal Research and Clinical Center of Physical-Chemical Medicine of Federal Medical Biological Agency, Moscow, Russia; 2 Moscow Institute of Physics and Technology (National Research University), Dolgoprudny, Russia; 3 Center for Precision Genome Editing and Genetic Technologies for Biomedicine, Federal Research and Clinical Center of Physical-Chemical Medicine of the Federal Medical and Biological Agency, Moscow, Russia; 4 Shemyakin-Ovchinnikov Institute of Bioorganic Chemistry, Russian Academy of Sciences, Moscow, Russia

**Keywords:** Genome informatics, Software, Standards, Medical genetics, Population genetics

## Abstract

Whole-exome sequencing is an attractive alternative to microarray analysis because of the low cost and potential ability to detect copy number variations (CNV) of various sizes (from 1–2 exons to several Mb). Previous comparison of the most popular CNV calling tools showed a high portion of false-positive calls. Moreover, due to a lack of a gold standard CNV set, the results are limited and incomparable. Here, we aimed to perform a comprehensive analysis of tools capable of germline CNV calling available at the moment using a single CNV standard and reference sample set. Compiling variants from previous studies with Bayesian estimation approach, we constructed an internal standard for NA12878 sample (pilot National Institute of Standards and Technology Reference Material) including 110,050 CNV or non-CNV exons. The standard was used to evaluate the performance of 16 germline CNV calling tools on the NA12878 sample and 10 correlated exomes as a reference set with respect to length distribution, concordance, and efficiency. Each algorithm had a certain range of detected lengths and showed low concordance with other tools. Most tools are focused on detection of a limited number of CNVs one to seven exons long with a false-positive rate below 50%. EXCAVATOR2, exomeCopy, and FishingCNV focused on detection of a wide range of variations but showed low precision. Upon unified comparison, the tools were not equivalent. The analysis performed allows choosing algorithms or ensembles of algorithms most suitable for a specific goal, e.g. population studies or medical genetics.

## Introduction

Copy number variations (CNVs) are variations of the number of copies of a DNA fragment in a population. According to a high-resolution CNV map composed using publicly available data, CNVs cover 4.8–9.5% genome^1^ and can be involved in both evolutionary adaptation and disease progression^2,3^. Due to the low resolution of chromosome microarray analysis (CMA), CNV detection mainly relies on next-generation sequencing (NGS) data^4,5^. Whole exome sequencing (WES) is a cost-effective and wide-spread technique primarily used for detection of small variants in coding regions of the genome. Due to its high sensitivity, the method is widely used in population studies (e.g. the Exome Aggregation Consortium^6^); also it is included in guides for identification of the genetic causes of many diseases^7,8^. WES data could also be used as an information resource to detect CNVs. However, WES has many features that impede accurate CNV detection. These include basic features (like capture step) and those originating from the PCR stages (problems with sequencing low complexity regions, dependence on GC content), directly affecting the over- and underrepresentation of target regions, which can be mistakenly interpreted as CNVs.

Multiple tools have been elaborated to detect CNVs in exome data; they mainly use the read depth-based strategy, in which the number of reads (read count, RC) mapped onto a fragment of interest is being evaluated^9,10^. These tools vary greatly at every step of the analysis, including read-depth distribution assumption, RC data normalization, and segmentation approach (Table 1). For example, ExomeDepth^11^ uses a beta-binomial model of read distribution and CANOES^12^ and exomeCopy^13^ apply negative binomial distribution due to errors in the course of sample preparation and hybridization. cn.MOPS^14^ utilizes a mixture of the Poissons model and Bayes approach. The EXCAVATOR2^15^ and CNVkit^16^ tools predict CNVs taking into account not only normalized RC of target regions but also that of the off-target ones. To improve the efficiency of CNV calling, CLAMMS^17^ and ExomeDepth also include a procedure of reference set optimization. CoNIFER^18^ performs systematic bias correction using singular decomposition and CODEX^19^ applies log-linear decomposition-based normalization. Both XHMM^20^ and FishingCNV^21^ use principal component analysis to reduce noise but FishingCNV applies circular binary segmentation (CBS) on the test sample and then performs the simple comparison of normalized coverage against background, while XHMM uses hidden Markov models on Z-RPKM (reads per kilo-base per million total reads) values. Moreover, some algorithms have other features: ExonDel^22^ and HMZDelFinder^23^ can only detect decrease in the number of copies in the genome, and PatternCNV^24^ and CONTRA^25^ are focused on the identification of exon-level CNVs. Among all tools, only DeAnnCNV^26^ is available online and, in addition to CNV calling, includes the variation annotation module. CNVkit, CODEX, EXCAVATOR2, ExomeDepth, ExonDel, PatternCNV can be considered as more universal for WES analysis, they are designed for both germline and somatic CNV calling.

**Table 1:** CNV calling tools included in the study.

Tool	Algorithm detail	Features (specifics)	Year
CANOES	Negative binomial distribution, regression-based normalization (GC-content), HMM	At least 15 samples, average targets 6, distance between targets 70 kb, average rate of CNV occurrence in the exome 10–8	2014
CLAMMS	GC-content and average depth normalization, custom reference set using kNN, mixture model, HMM	0.3 < GC < 0.7, mappability > 0.75	2015
cn.MOPS	GC-content and sample normalization, mixture Poissons model and Bayes approach	At least 6 samples Minimum segments 5	2012
CNVkit	In-target and off-target regions, bias (GC-content, repeat-masked fraction, target density) correction using rolling median, CBS	Exclude poor mappable regions	2016
CODEX	Log-linear decomposition-based normalization, Poisson likelihood-based segmentation	0.2 < GC < 0.8 Target length > 20 bp, median target coverage > 20 × , mappability > 0.9	2015
CoNIFER	Singular value decomposition normalization, ± 1.5 SVD-ZRPKM threshold	At least 50 samples Probes with median RPKM across samples > 1, samples with a standard deviation of SVD-ZRPKM < 0.5	2012
CONTRA	Base-level log-ratios, GC-content, library-size correction, calling region significant based on normal distribution, CBS for large variation	Include regions at least 10-bp long with coverage > 10	2012
DeAnnCNV	Web-server, GC-normalization, HMM of log read counts ratio	CNV evidence threshold > 80	2015
EXCAVATOR2	In and off-target regions, 3-step normalization (GC-content, mappability, region length) segmentation with shifting level model, FastCall algorithm	Read mapq > 1 Min number of targets in CNV 4	2016
exomeCopy	Negative binomial distribution, HMM using background read depth and positional covariates (GC-content, length)	mapq > 1, overlap to include read into region—1 bp, median value for background, transition probability to CNV 1e-4 Transition probability to normal state 0.05	2011
ExomeDepth	Beta-binomial distribution, optimized reference set, HMM	Read mapq > 20, max distance between target border and the middle of paired read to include read into region 300 bp Transition probability to CNV 0.0001 Expected CNV length 50 kb	2012
ExonDel	Deletion in exome or genes of interest, GC-content median correction, calling by comparing to median depth within the gene	Read mapq > 20, base quality > 20, min percent of covered bp for each exon 0.1, max number of exons in CNV 9	2014
FishingCNV	PCA of RPKM, CBS test sample, comparing segment coverage against control set distribution	Read mapq > 15 Base quality 10, RPKM > 3 FDR adjusted *p*-value 0.05	2013
HMZDelFinder	Only deletion, exon and sample filtering, call region with RPKM < 0.65 as deletion, AOH filtering based on VCF, prioritization based on Z-score	Mean RPKM > 7 across samples, deletion frequency < 0.5% Exclude 2% samples with the highest number of deletion	2017
PatternCNV	Log2-transformed RPKM standardization, average and variability pattern training from control samples, smooth bin within exon	Bin size 10 mapq > 20	2014
XHMM	Gaussian distribution, PCA normalization, HMM	At least 50 samples, 0.1 < GC < 0.9, 10 bp < target < 10 kbp, mean coverage > 10 × across all samples, average targets 6, distance between targets 70 kb, average rate of CNV occurrence in the exome 10–8	2012

There are several studies focusing on comparison of sets of 3–6 existing CNV calling algorithms^27–29^. However, there are no works that would compare larger sets of currently available tools. Also, the criteria for estimation of their efficiency on validation data could be improved. Existing studies consider only well-known tools (XHMM, CONIFER, ExomeDepth, CONTRA, exomeCopy) and use different overlap criteria to confirm predictions. One group used CNVnator^30^ calls from whole-genome sequencing (WGS) data as a CNV set standard; the other used calls obtained from CMA, including custom design arrays.

In addition to in-house generated data, public data is also used to evaluate algorithm performance. Since CNVs have been acknowledged as a natural part of the human genome, more than 70 studies have been performed to identify CNVs in the human genome. The most widely-used CNV call sets are: (1) the study of Conrad et al. (2010), in which^31^ a set of 20 comparative hybridization arrays with 2.1 million probes to identify CNVs over 500 bp long has been used; (2) structural variations obtained during the pilot and/or phase 3 of the 1000 Genomes Project, predicted from the whole-genome data by 19 different algorithms (read-depth, read-pair, split-read, assembly-based)^32–34^; (3) and high-confidence CNV calls from NA12878 sample supported by multiple signals using svclassify^35^. Specific features of CNV set formation, for example, accuracy of prediction from WGS or low resolution and choice of the reference sample in case of array technology, do not allow to obtain a comprehensive spectrum of variations and identify all the advantages and limitations of a CNV calling tool.

To perform a unified comparative analysis: (1) we chose NA12878 as one of the most characterized samples of the Genome in a Bottle project; (2) we used exon as a minimal unit for comparison, (3) we constructed the set of CNV and non-CNV exons based on available CNV sets for the NA12878 using Bayes model, and (4) we evaluated the performances of 16 existing germline CNV tools (Table 1) using the same reference set.

## Methods

### Study data

The collection of exome data mapped to GRCh37 decoy reference genome (hs37d5.fa) in the BAM format was downloaded from Phase 3 of the 1000 Genomes Project (hg19) (detailed description of samples is provided in Supplementary Table S1). All alignment data were processed by the standard data pre-processing protocol: sorting, filtering (deleting the reads with MAPQ > 10) with SAMTools v.0.1.19, and removing PCR duplicates by Picard MarkDuplicates v. 2.5.0. The average coverage for the data was 104.3X, and the average fraction of target regions covered with at least 10X and 20X were 89.9% and 83.1%, respectively.

To construct validation set structural variations previously detected in the NA12878 sample were received from the sixteen peer-reviewed research studies^31–47^; experiment design and data processing procedures for each of them are described in Supplementary Table 2. If calls were not available for hg19 genome build we converted their coordinates using UCSC LiftOver. To operate at the exon level, we considered an exon as an exon with copy number variation (CNV-exon) if the CNV region spans at least 50% of the exon. The set of known exons was obtained from GENCODE comprehensive gene annotation Release 19 (GRCh37.p13); to construct a set of non-overlapping exons per gene, the *R GenomicFeatures* package was used.

### Parameters of CNV calling tools used

The CNV calling from exome data were performed by 16 tools (Table 1). As a reference set for the NA12878 sample we used the top 10 most correlated samples according to recommendations^11^ (function *select.reference.set* implemented in the ExomeDepth 1.1.10 software package). Most tools were run with default parameters, all changes in standard pipelines for the rest of the tools are described above. For GC-content calculation implemented in some algorithms, GRCh37 decoy reference genome (hs37d5.fa) was used.

During the CONIFER normalization step, two singular value decomposition components were removed based on the inflection point of the scree plot for our data; for calling, threshold value 0.95 was used due to the small coverage variability for correlated samples. ExomeDepth and CLAMMS were applied without their reference set selection step; also all CNVs with non-Phred quality score < 0 identified by CLAMMS were filtered off. To predict CNV in a test sample by cn.MOPS, a pipeline for exome sequencing data (*exomecn.mops*) was used on the preliminarily calculated read count matrix. Since longer calls are more reliable, we consider ExonDel results found by a moving window with a maximum of the available number of exons (9). CNV calling with HMZDelFinder was performed without theAOH analysis. FishingCNV and PatternCNV calls with *p* value > 0.05 were excluded.

### Performance evaluation

Analysis of the existing tools was performed only on autosomal chromosomes with respect to the characteristics of predicted CNVs (number of calls, size, total length, target regions), concordance between algorithms, and efficiency of the latters. To assess the prediction accuracy of an algorithm, we constructed the validation set of CNV-exons for NA12878 based on the previous research using Bayes model (See “Construction of a standard CNV-exon set based on Bayesian estimation” section). For correct estimation, we considered only those exons that are included in the target design and our validation set. Since the data is inherently unbalanced we used the following metrics for evaluation of a tool:

$$Recall = \frac{{True\;Positive}}{{True\;Positive + False\;Negative}},$$

$$Precision = \frac{{True\;Positive}}{{True\;Positive + False\;Positive}},$$

$$F1 = \frac{{Recall*Precision}}{{Recall + Precision}}.$$

## Results

### Moderate concordance among CNV validation sets

A comprehensive validation set is needed to adequately compare existing methods. Due to the wide range of sizes and types of structural variation, the development of such is challenging. Although the NA12878 sample is one of the standards for benchmarking of multiple callers, a gold-standard CNV set doesn’t exist. The recent set of high-confidence CNVs (*svclassify*) proposed by the GIAB Consortium includes only deletion events and besides it only spans 184 exons. Therefore, we collected CNV call sets for the NA12878 sample from previous studies (Supplementary Table S2).

**Figure 1:**
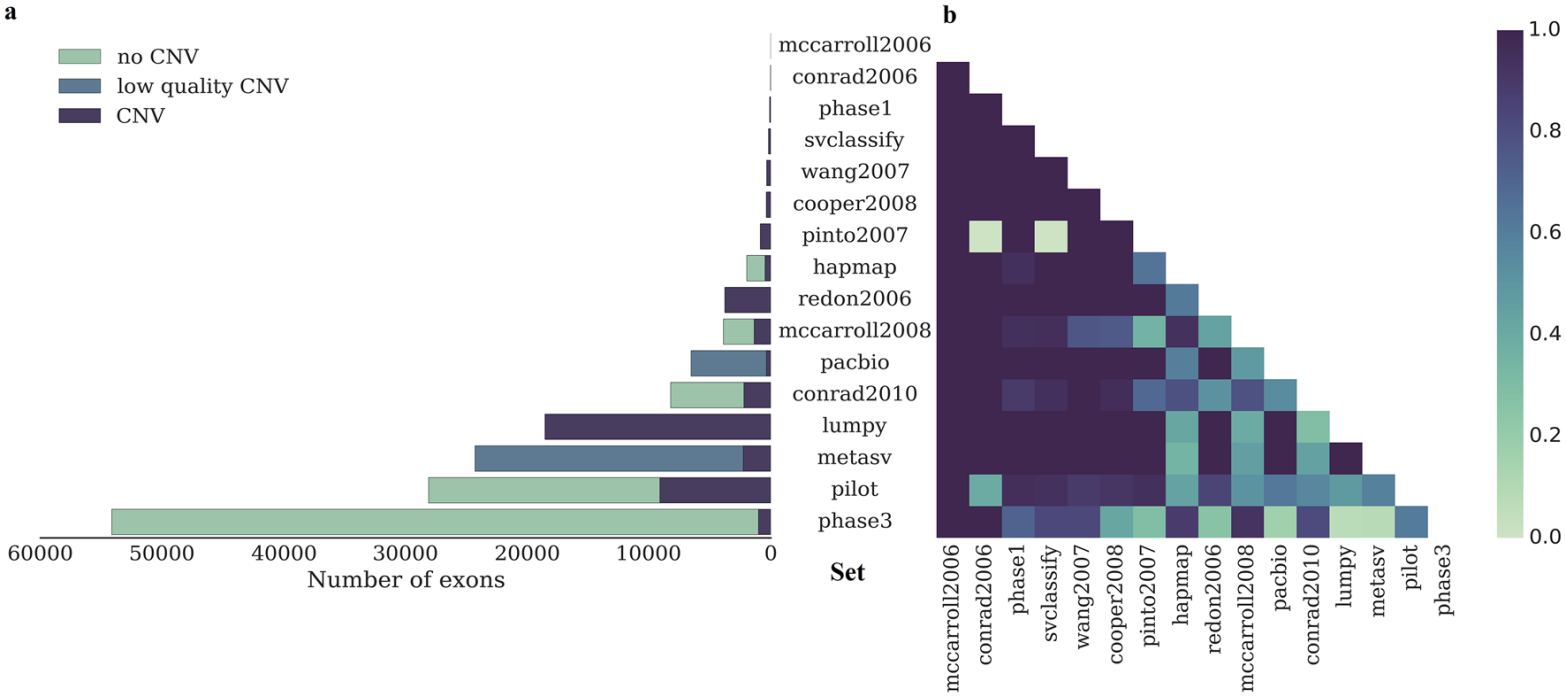
Size of validation sets for the NA12878 sample (**a**) and rate of exons with equal states (both CNV or both non-CNV) for each pair of sets (**b**).

To perform the pairwise comparison of the 16 CNV sets we evaluated the set size and the fraction of exons with the same status (CNV or non-CNV) in shared exons of any two validation sets (Fig. 1, Supplementary Table S3). Early studies^36–41^ (Supplementary Table S2; the *mccarroll2006*, *conrad2006*, *wang2007*, *pinto2007*, and *coper2008* sets) detected a small number of CNVs but almost all of them were well supported by later research. The sets obtained by the integration signals from multiple technologies (e.g. *svclassify, metasv)* also showed a high level of concordance with other sets.

Another problem of the available sets is their limitation to identify false positives. The 5 sets (*mccarroll2008*, *conrad2010*, *hapmap*, *pilot*, and *phase3*) contained information about true non-CNV exons, their pairwise comparison at the exon level is shown in Table 2.

**Table 2:** Pairwise comparison of sets with validated non-CNV exons and the GIAB high-confidence CNV set (*svclassify*): elements on the main diagonal indicate the set size, for other elements the denominator of a fraction is the number of shared exons in the sets and the numerator is the number of exons with the same state.

	*svclassify*	*hapmap*	*mccarroll2008*	*conrad2010*	*pilot*	*phase3*
*svclassify*	184					
*hapmap*	41/41	1970				
*mccarroll2008*	41/43	1211/1282	3875			
*conrad2010*	81/85	975/1228	1192/1514	8230		
*pilot*	164/174	406/925	651/1276	1364/2431	28104	
*phase3*	103/124	1355/1522	2272/2450	3142/3847	3808/6176	54137

Overall, the number of CNV-exons varied greatly among the sets; on average, they overlapped by 40%. These results can be explained by differences in the approaches, reference sets, and criteria used to call CNVs in different studies. Such differences in the validated exons cast doubt on the correctness of use of only one of these sets for the comparative analysis of CNV tools. Therefore, we made an attempt to generate our own validation set using available data.

### Construction of a standard CNV-exon set based on Bayesian estimation

Since available CNV sets describe different genome regions, and for some of them true non-CNV are not defined, it is impossible to formulate the majority rule for any exon. Assuming that all sets are independent, we considered that in conditions of conflicting and incomplete information, the Bayes estimator would be the most appropriate approach for making a decision. In Bayes estimation, the unknown parameter $\theta$ is viewed as random and the model is specified in term of the conditional probability density function $f(x|\theta )$ and prior distribution $\pi \left( \theta \right)$ on $\theta$. By Bayes rule, the posterior distribution of given some measurement x:

1 $$\pi \left( {\theta {\text{|}}x} \right) \propto ~f\left( {x{\text{|}}\theta } \right)\pi \left( \theta \right).$$

The quality of the estimate is measured by loss function $L\left( {\theta ,\delta } \right)$ and estimator $\delta$ which minimizes the expected posterior loss $E[L\left( {\theta ,\delta } \right)|x]$ for each x is called Bayes estimator^48^.

To construct the validation set we wanted to rank exons based on their estimation of being CNV-exon and consider the most highly ranked as CNV. Let *i*th exon is characterized by *n* number of CNV sets and *x* sets described *i*th exon as CNV-exon. Suppose that $x\sim Binomial\left( {n,\theta } \right),~~\theta \sim ~Beta\left( {\alpha ,\beta } \right)$, where $\theta$ is the probability of being CNV-exon, then posterior distribution

2 $$\pi \left( {\theta |x} \right) = \left( {\begin{array}{*{20}c} n \\ x \\ \end{array} } \right)\theta ^{x} \left( {1 - \theta } \right)^{{n - x}} \theta ^{{\alpha - 1}} \left( {1 - \theta } \right)^{{\beta - 1}} \frac{{\Gamma \left( {\alpha + \beta } \right)}}{{\Gamma \left( \alpha \right)\Gamma \left( \beta \right)}}~\sim Beta\left( {\alpha + x,\beta + n - x} \right)~.$$

The most common estimation problem is squared error loss due to its simplicity and convenience. It is characterized by high sensitivity to outliers and the same magnitude for positive and negative errors. However, to focus on confident CNV-exons especially under the limited number of sets including data about true diploid genome regions we needed to penalize errors differently. Therefore we considered an asymmetric piecewise linear loss function^49^

3 $$L_{{t_{1} t_{2} }} \left( {\theta ,d} \right) = \left\{ {\begin{array}{*{20}l} {t_{2} \left( {\theta - d} \right)~} \hfill & {if\;\theta > d,} \hfill \\ {t_{1} \left( {d - \theta } \right)} \hfill & {otherwise.} \hfill \\ \end{array} } \right.$$

Then the expected posterior loss may be written

4 $$E[L_{{t_{1} t_{2} }} \left( {\theta ,d} \right){\text{|}}x{\text{]}} = t_{1} \mathop \smallint \limits_{0}^{d} \left( {d - \theta } \right)\pi \left( {\theta {\text{|}}x} \right)d\theta + t_{2} \mathop \smallint \limits_{d}^{1} \left( {\theta - d} \right)\pi \left( {\theta {\text{|}}x} \right)d\theta$$

Minimizing this expression with respect to *d* we get that Bayes estimator is $\frac{{t_{2} }}{{t_{1} + t_{2} }}$ fractile of posterior distribution (Eq. 5):

5 $$\begin{aligned} & 0 = t_{1} \left( {d - d} \right)\pi \left( {d{\text{|}}x} \right) + t_{1} \mathop \smallint \limits_{0}^{d} \pi \left( {\theta {\text{|}}x} \right)d\theta - t_{2} \left( {d - d} \right)\pi \left( {d{\text{|}}x} \right) - t_{2} \mathop \smallint \limits_{d}^{1} \pi \left( {\theta {\text{|}}x} \right)d\theta \\ & t_{1} \mathop \smallint \limits_{0}^{d} \pi \left( {\theta {\text{|}}x} \right)d\theta - t_{2} \left( {1 - \mathop \smallint \limits_{0}^{d} \pi \left( {\theta {\text{|}}x} \right)d\theta } \right) = 0 \\ & I_{d} \left( {\alpha + x,\beta + n - x} \right) = \frac{{t_{2} }}{{t_{1} + t_{2} }}, \\ \end{aligned}$$

where $I\left( {a,b} \right)$ is the regularized incomplete beta function.

To evaluate the parameters of a priori distribution, we used the stringent CNV map of the human genome constructed by Zarrei and co-authors^1^. Using the data on the European population, we constructed the probability distribution of the presence of CNVs in 21,242 exons that are crossed by the CNV regions. Probability distribution was fit using the maximum likelihood method (python function *scipy.stats.beta.fit* with *location* = 0 and *scale* = 1), which yielded $\alpha = 0.33$ and $\beta = 0.93$.

The penalties were chosen out of concern that overestimation of the parameter was worse than underestimation. For simplicity, we defined the coefficients as the maximum number of sets that are available to characterize exon as CNV (*t*_1_ = 16) and non-CNV (*t*_2_ = 5). To solve the Eq. (5), the *special.betaincinv* function of the Python SciPy version 1.0.0 was used.

**Figure 2:**
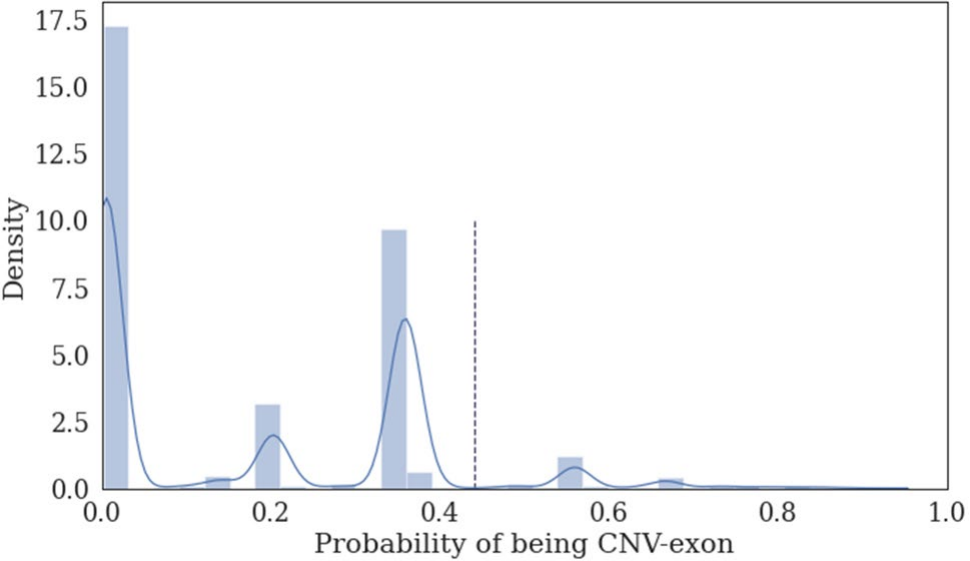
Distribution of the probability estimation. Threshold for exon classification is indicated by a dotted line.

The distribution of the estimated exon’s probability of being CNV is presented in Fig. 2. As we expected, exons are divided into groups depending on their representation in CNV sets. The highest left peak corresponded to exons described only as non-CNV. Data about exons with parameter value in the 0.2–0.4 range were the most contradictory, with a slight preponderance in favor of two copy number region or CNV; exons described by only one set were also ranked low. On other exons, we observed a prevalence of sets confirmed CNV. A cutoff of 0.45 for selecting confident CNV-exons based on their estimates was chosen to provide 95% accuracy on a set of 225 CNV-exons validated using PCR. Thus, our validation set for NA12878 contained 6853 CNV-exons and 103,197 non-CNV exons.

To compare the internal standard and the initial data, we estimated the percentage of CNVs in any CNV set included in our standard. The recent set of high-quality CNVs formed in the framework of the GIAB project and experimentally validated variants from Conrad and McCarroll studies from 2006 were fully integrated into the standard. The constructed validation set contains above 79% of CNV-exons discovered from WGS (The 1000 Genome Project, FDR < 5% by orthogonal validation methods), SNP-array data (hapmap, wang2007, pinto2007, cooper2008). Variations from mccarroll2008, redon2006 and conrad2010 discovered by genotyping array and CGH array respectively were presented only a half in the standard, the remaining CNV-exons were singleton (presented only in one CNV set) and insufficiently confirmed. The same situation was observed for predictions based on long reads or integration of different signals (paired-read, split-read, read depth). The observed relationships between sets allow us to consider the standard as reliable for benchmarking analysis.

### Evaluation of algorithms

To take into account the genome variability and reduce the number of false-positive calls, we chose 10 exomes well-correlated by coverage with NA12878 as a reference sample set and run 16 germline CNV calling tools on these exome data.

#### Differences in count and size of predicted CNVs

The algorithms differed by an order of magnitude in terms of both the number of predicted variations and the number of affected target regions (Table 3). The highest number of variations were predicted by FishingCNV (1210 CNV) and exomeCopy (845 CNV); the lowest, by a web-tool DeAnnCNV (2 CNV). CONTRA, EXCAVATOR2, ExomeDepth, and PatternCNV identified about 200–300 variations; in the case of CONTRA and PatternCNV these were single-exon CNVs. Other algorithms detected an average of 26 variations. Most algorithms preferentially detected deletions over duplications, which can be explained by peculiarities of the data and better distinguishes between decreases relative to diploidy than increases. The total length of CNVs ranged from 50 kb to 1304 Mb, which exceeds the known fraction of CNVs in the human genome. This indicates the need to filter the calls produced by some tools, in particular, by FishingCNV and exomeCopy.

**Table 3:** Description of predicted CNVs.

Algorithm	Number of CNVs	Deletion	Duplication	Target*	Mean of targets**	Total length of CNVs, kb	Mean CNV length, kb	CNV size range, kb
CANOES	16	16	0	306	19	3583.4	51.5	0.4–2056.1
CLAMMS	34	13	21	62	2	122.6	3.6	0.1–77.7
cn.MOPS	38	29	9	276	7	2207.9	21.5	1.8–886.8
CNVkit	16	16	0	309	19	4194.4	262.1	0.5–1347.4
CODEX	97	56	41	885	9	8314.7	85.7	4.4–117.3
CoNIFER	12	12	0	124	10	343.5	15.2	2.5–185.1
CONTRA	329	244	85	329	1	135.8	0.24	0.1–16.8
DeAnnCNV	2	1	1	7	3	121.7	60.8	4.4–117.3
EXCAVATOR2	236	183	53	5002	21	40359.8	71.6	1.4–2741.6
exomeCopy	845	790	55	91059	108	1,304,244.3	1543.5	1.2–40,705.9
ExomeDepth	199	151	48	1057	5	5018.7	4.1	0.1–693.0
ExonDel	17	17	0	529	31	849.5	50.0	6.0–356.1
FishingCNV	1210	815	395	54434	45	1,115,772.5	922.1	0.1–26,762.8
HMZDelFinder	7	7	0	15	2	99.2	14.2	0.1–63.5
PatternCNV	243	233	10	254	1	51.2	0.2	0.0–1.63
XHMM	11	8	3	211	16	1069.6	40.3	5.2–534.4

*Target, number of target regions covered by all predicted CNVs.

**Mean of target, average number of target regions covered by a CNV.

**Figure 3:**
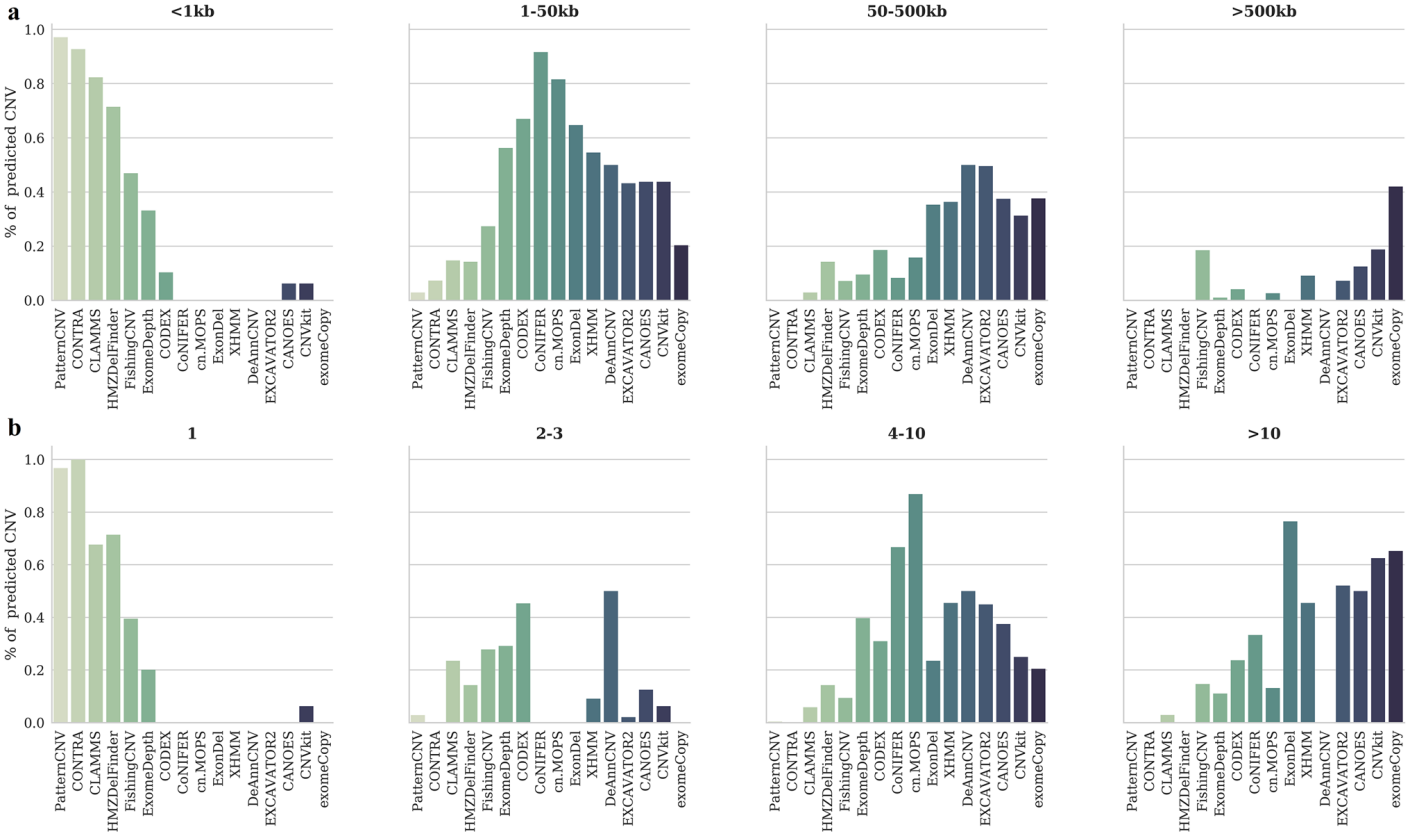
Distribution of predicted CNVs over length (**a**) and number of targeted regions (**b**).

We noticed that CNV tools can be divided into groups based on the length of identified CNVs. The algorithms Most algorithms detect CNVs within 1–50 kb with an average number of targeted regions equal to 7 (Fig. 3).

**Figure 4:**
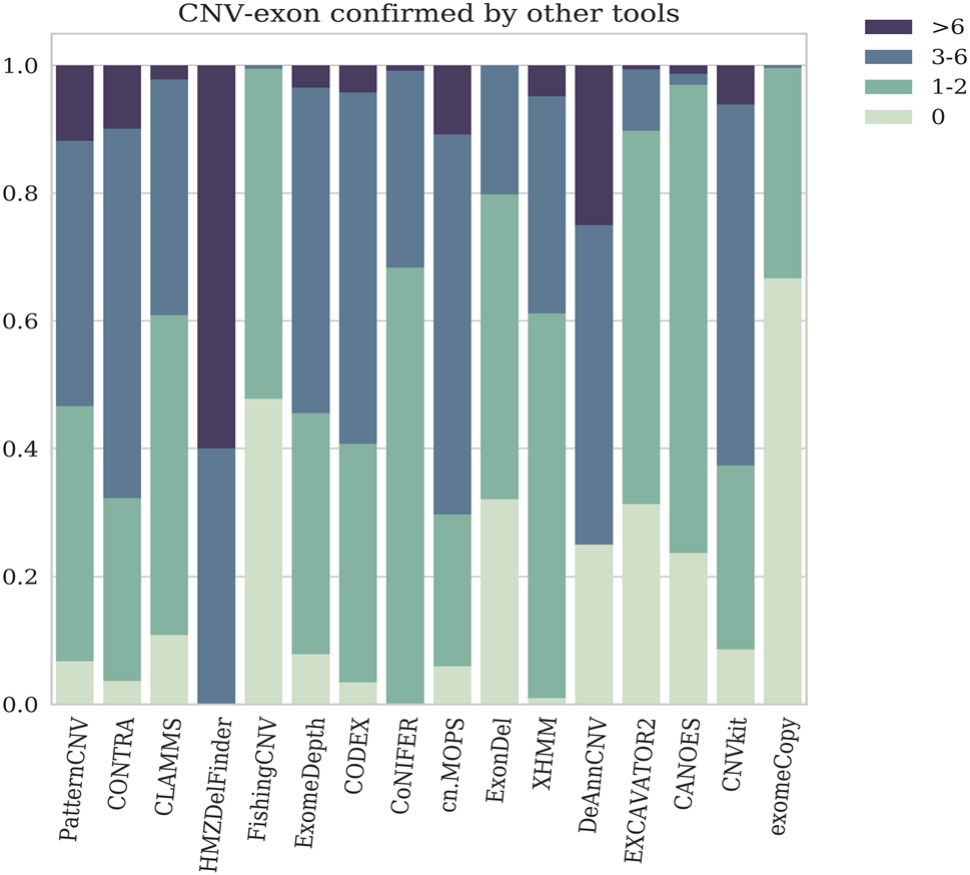
Fraction of predicted CNV-exons confirmed by 1–2, 3–6, or over 6 tools. The order of the tools is determined by the length of predicted CNVs: from small to large.

**Figure 5:**
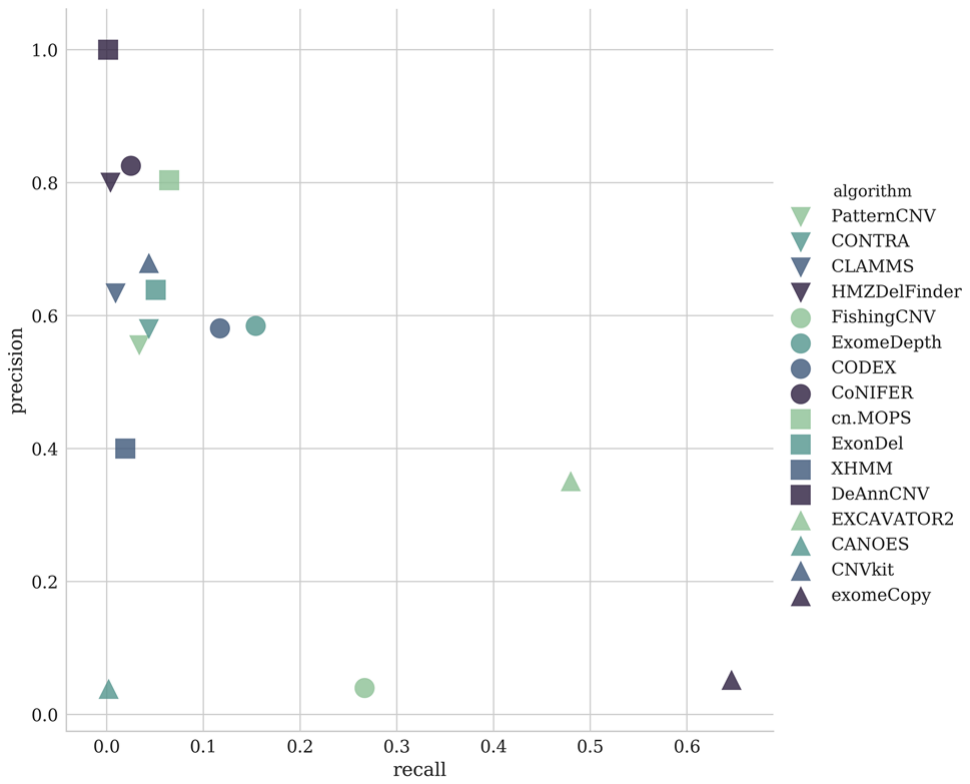
Recall and precision of algorithms for CNV identification using whole-exome sequencing data.The order of the algorithms is determined by the length of predicted CNVs: from small to large.

Variations of less than 1 kb were found by nine tools (ExomeDepth, CONTRA, CANOES, CLAMMS, CNVkit, CODEX, FishingCNV, HMZDelFinder, and PatternCNV); moreover, for CLAMMS, CONTRA, PatternCNV, FishingCNV, and HMZelFinder such variations account for a large fraction of identifications. CNVkit, CODEX, CANOES, EXCAVATOR2, and FishingCNV are among the few algorithms that detect both small CNVs from 2 to 3 target regions and long variations (over 1 Mb).

#### Concordance between CNV calling tools

Concordance check between tools was performed at the exon level. About 70,000 CNV-exons were singleton, i.e. detected by a single tool (Supplementary Table S4), and the main portion was called by exomeCopy and FishingCNV. The other predictions of these two algorithms mostly intersected with EXCAVATOR2. For ExonDel, CANOES, EXCAVATOR2, and DeAnnCNV, 25–30% of predicted CNV-exons were unique, while for the rest of the tools more than 90% predicted CNV-exons were confirmed by the others. The best confirmation was observed for HMZDelFinder and CONIFER calls (Fig. 4, Supplementary Table S6) with zero unique CNV-exons. Despite the high-level matching of calls with at least one tool, we observed a low number of common CNV-exons called by multiple tools (3 and more). The maximum available overlapping spanned 2 exons and included 11 algorithms (PatternCNV, CODEX, ExomeDepth, XHMM, FishingCNV, CONTRA, CANOES, HMZDelFinder, EXCAVATOR2, cn.MOPS, CNVkit). We also performed pairwise comparison of calling tools (Supplementary Table S6). CODEX and ExomeDepth showed the best concordance with almost 33% of common CNV-exons. exomeCopy and FishingCNV had 24% of concordant CNV-exons but unlike previous ones they practically do not intersect with other algorithms. On average, a CNV calling tool had some agreement (> 5%) with 3–4 others. The worst similarity was observed for CANOES (no more than 1%). cn.MOPS, CONTRA, PatternCNV, CoDEX and ExomeDepth paired with each other showed a moderate concordance level (10–21%).

#### Accuracy of CNV calling tools

We estimated the efficiency of CNV calling tools using the generated set of CNV and non-CNV exons (Supplementary Table S7, Fig. 5). Among all algorithms, CANOES turned out to be the worst with 3.9% precision and 0.2% recall. The highest recall was demonstrated by exomeCopy: it identified 65% of exons of our standard. On the other hand, the algorithm predicted many false-positive events (about 95% identifications). Second best in terms of the number of predictions was the FishingCNV algorithm. It showed 27% recall at 4% precision value. Sensitivity of other algorithms did not exceed 16%, although they differed considerably in terms of precision of exon identification: from 40% for XHMM to 100% for DeAnnCNV. High precision values were also obtained for CONIFER (82%), HMZDelFinder (80%), and cn.MOPS (78%). EXCAVATOR2 is the most balanced one, with F1-score 0.41. ExomeDepth (F1-score 0.24) and CODEX (F1-score 0.19) identified lower number of variants, but were less prone to false identifications.

Therefore, all algorithms have their own focus. Most algorithms analyzed are fit for a limited search of CNVs involving 1–7 exons with the rate of false-positive results not exceeding 50%. At the same time, EXCAVATOR2, exomeCopy, and FishingCNV producing 30–60% unique predictions are tailored for the search of a wide range of variations but are characterized by low precision.

## Discussion

CNV is an important type of structural variation, accurate detection and interpretation of which are essential for both population studies, medical genetics, evolution, and cancer research. Chromosomal microarray analysis often is limited by array resolution and detects mostly the major rearrangements. WGS and WES, in turn, can detect all levels of CNVs and are of interest in clinical practice, especially WES, which is the primary diagnostic test for many orphan diseases, spectrum disorders and syndromes. Despite the development of many CNV calling tools, detection of this type of variation remains challenging. The reason for this is not only poor sequencing efficiency in regions with low sequence complexity or regions with high GC-content, but also the challenge to construct a true set of CNVs for adequate evaluation of applied methods.

Using a sample from the “Genome in the Bottle” project as an example, we considered the contradictions between available CNV call sets arising from differences in the resolution of detection methods, choice of the reference pool (from one to several dozens of samples), and analysis tools. Since no set covers all CNVs to the full, we used a stringent CNV map of the human genome as prior knowledge and with the Bayes approach ranged the exons by the probability of being CNV-exons. Thus, we constructed an exon-level CNV set for the NA12878 sample which includes about 110 thousand exons and can be used for an independent evaluation of tools on exome data.

Comparative analysis of 16 germline CNV calling tools showed that each algorithm has its certain range of detected lengths: the minimum possible size is an exon as predicted by CONTRA or PatternCNV, and the maximum size is more than 1 Mb, as observed in CNVkit calls. Most algorithms call CNVs up to 100 kb in length and span 4–10 exons. Due to the nature of the sequencing data, tools preferentially detected loss of genetic material over its gain.

We observed low concordance between the results produced by different tools. In addition to the differences in models applied for CNV calling, one of the possible reasons for the situation can be the characteristics of the exons. In particular, part of the CNV-exon singletons was obtained for exons with extreme values of GC content and mappability, which are filtered off in some of the algorithms (Table 1).

Algorithms are most effective in detection of variation from 1 kb, it could be a feature of calling model fitting: choice of train data and evaluation criterion for variant (e.g. number of targets). Also we showed differential focus of tool performance on precision or recall. Most algorithms identify a small number of variations yet with satisfactory precision (about 70%). This group of algorithms can be of use in population studies. In case of clinical diagnostics, there is a need to identify as many variations as possible with exome sequencing even if at the expense of precision. Later on the variants will be evaluated based on the clinical presentation, de novo appearance compared to parents and joint effect of revealed variants on the phenotype—thus filtering the false-positive variants. Also, in case studies, additional validation of individual candidate CNVs using cheaper techniques is possible, which decreases the false-positive rate and enhances the importance of minimization of false-negative outputs. This condition is met by only three algorithms: EXCAVATOR2, exomeCopy, and FishingCNV with recall over 26%. Therefore, part of CMA performed in frames of diagnostics of hereditary disorders can be replaced with primary CNV calling from exome. The minimum precision threshold should be chosen depending on the aims of the analysis and in the case of medical applications, on the group of disorders under investigation.

Limitations in the applicability of existing algorithms described in the article (number of CNV, expected length, calling efficiency) should be considered when using CNV calling. In the case of detecting small variations (1 exons), PatternCNV and CONTRA are practically equivalent in terms of the number of CNVs and prediction accuracy. The CNV calls intersection increases precision to 73%. At the main range (1–100 kb) we recommend using cn.MOPS if the goal is low false positive rate (20%) and EXCAVATOR for more sensitive CNV calling.

The findings of this study have to be seen in light of some limitations. The first is the analysis was conducted on only one sample. Since the development of a CNV benchmark set is a rather laborious process, the choice of samples for evaluating the algorithm's efficiency is limited. Expanding the set of samples would allow researchers to fully evaluate the characteristics of the predicted CNV and investigate performance results under different sequencing depths. The second is the formation of the validation set at the exon level. Such a description does not allow us to assess the efficiency of identifying CNV of different lengths due to the complexity of assigning “true” CNV-exon to one or another group. The third limitation concerns the fact that uniform conditions may not be optimal for algorithms. For example, the minimum number of samples required for quality identification or using only the default parameters. Comparison of the best performances is of particular interest for understanding the capabilities of CNV calling tools. Moreover, we think this may be a good practice in the future to set the parameters depending on the available data or alternatively use ensemble models, thereby increasing the detection efficiency.

## Supplementary Information

Supplementary Tables.

## Data Availability

The code of the analysis is available at https://github.com/bioinformatics-IBCH/Comparison-study-of-germline-CNV-calling-tools.
