# Supplementary material for: Benchmarking germline CNV calling tools from exome sequencing data
Source: Sci Rep. 2021 Jul 13;11:14416. doi: 10.1038/s41598-021-93878-2 (PMC8277855; doi:10.1038/s41598-021-93878-2)
Supplement: Supplementary file 1 — Supplementary Tables. [file 41598_2021_93878_MOESM1_ESM.pdf]

Benchmarking germline CNV calling tools from exome sequencing data

Veronika Gordeeva*, Elena Sharova , Konstantin Babalyan, Rinat Sultanov, Vadim Govorun and Georgij Arapidi

Supplementary Table S1. Samples info

Sample	Population	Sex	Center*	Capture Technology	Platform	Correlation with NA12878
NA12878	CEU	female	BI	Agilent SureSelect_All_Exon_V2	ILLUMINA HiSeq 2000	1,000
NA06989	CEU	female	BI	Agilent SureSelect_All_Exon_V2	ILLUMINA HiSeq 2000	0,978
NA07347	CEU	male	BI	Agilent SureSelect_All_Exon_V2	ILLUMINA HiSeq 2000	0,965
NA07051	CEU	male	BI	Agilent SureSelect_All_Exon_V2	ILLUMINA HiSeq 2000	0,955
NA18999	JPT	female	BI	Agilent SureSelect_All_Exon_V2	ILLUMINA HiSeq 2000	0,954
NA18959	JPT	male	BI	Agilent SureSelect_All_Exon_V2	ILLUMINA HiSeq 2000	0,954
NA18960	JPT	male	BI	Agilent SureSelect_All_Exon_V2	ILLUMINA HiSeq 2000	0,949
NA06986	CEU	male	BI	Agilent SureSelect_All_Exon_V2	ILLUMINA HiSeq 2000	0,939
NA11843	CEU	male	BI	Agilent SureSelect_All_Exon_V2	ILLUMINA HiSeq 2000	0,882
NA12340	CEU	male	BI	Agilent SureSelect_All_Exon_V2	ILLUMINA HiSeq 2000	0,880
NA12761	CEU	female	WUGSC	NimbleGen SeqCap_EZ_Exome v3	ILLUMINA HiSeq 2000	0,880
NA19159	YRI	female	WUGSC	NimbleGen SeqCap_EZ_Exome v3	ILLUMINA HiSeq 2000	0,862
NA19206	YRI	female	WUGSC	NimbleGen SeqCap_EZ_Exome v3	ILLUMINA HiSeq 2000	0,853
NA19131	YRI	female	WUGSC	NimbleGen SeqCap_EZ_Exome v3	ILLUMINA HiSeq 2000	0,852
NA12718	CEU	female	WUGSC	NimbleGen SeqCap_EZ_Exome v3	ILLUMINA HiSeq 2000	0,851
NA12249	CEU	female	WUGSC	NimbleGen SeqCap_EZ_Exome v3	ILLUMINA HiSeq 2000	0,838
NA19138	YRI	male	WUGSC	NimbleGen SeqCap_EZ_Exome v3	ILLUMINA HiSeq 2000	0,837
NA18981	JPT	female	WUGSC	NimbleGen SeqCap_EZ_Exome v3	ILLUMINA HiSeq 2000	0,833
NA11840	CEU	female	WUGSC	NimbleGen SeqCap_EZ_Exome v3	ILLUMINA HiSeq 2000	0,833
NA18973	JPT	female	WUGSC	NimbleGen SeqCap_EZ_Exome v3	ILLUMINA HiSeq 2000	0,833
NA18966	JPT	male	WUGSC	NimbleGen SeqCap_EZ_Exome v3	ILLUMINA HiSeq 2000	0,832
NA10847	CEU	female	WUGSC	NimbleGen SeqCap_EZ_Exome v3	ILLUMINA HiSeq 2000	0,831
NA19152	YRI	female	WUGSC	NimbleGen SeqCap_EZ_Exome v3	ILLUMINA HiSeq 2000	0,830
NA12751	CEU	female	WUGSC	NimbleGen SeqCap_EZ_Exome v3	ILLUMINA HiSeq 2000	0,827
NA19153	YRI	male	WUGSC	NimbleGen SeqCap_EZ_Exome v3	ILLUMINA HiSeq 2000	0,827
NA19223	YRI	male	WUGSC	NimbleGen SeqCap_EZ_Exome v3	ILLUMINA HiSeq 2000	0,819
NA18967	JPT	male	WUGSC	NimbleGen SeqCap_EZ_Exome v3	ILLUMINA HiSeq 2000	0,819
NA12760	CEU	male	WUGSC	NimbleGen SeqCap_EZ_Exome v3	ILLUMINA HiSeq 2000	0,815
NA12717	CEU	female	WUGSC	NimbleGen SeqCap_EZ_Exome v3	ILLUMINA HiSeq 2000	0,814
NA06994	CEU	male	WUGSC	NimbleGen SeqCap_EZ_Exome v3	ILLUMINA HiSeq 2000	0,810
NA18970	JPT	male	WUGSC	NimbleGen SeqCap_EZ_Exome v3	ILLUMINA HiSeq 2000	0,809
NA07037	CEU	female	BGI	NimbleGen SeqCap_EZ_Exome_v2	ILLUMINA HiSeq 2000	0,384
NA12347	CEU	male	BGI	NimbleGen SeqCap_EZ_Exome_v2	ILLUMINA HiSeq 2000	0,378
NA11893	CEU	male	BGI	NimbleGen SeqCap_EZ_Exome_v2	ILLUMINA HiSeq 2000	0,376
NA12413	CEU	male	BGI	NimbleGen SeqCap_EZ_Exome_v2	ILLUMINA HiSeq 2000	0,375
NA12775	CEU	male	BGI	NimbleGen SeqCap_EZ_Exome_v2	ILLUMINA HiSeq 2000	0,375
NA12827	CEU	male	BGI	NimbleGen SeqCap_EZ_Exome_v2	ILLUMINA HiSeq 2000	0,371
NA19239	YRI	male	WUGSC	NimbleGen SeqCap_EZ_Exome v3	ILLUMINA HiSeq 2000	0,232
NA12044	CEU	female	BCM	HSGC VCRome	ILLUMINA HiSeq 2000	0,231
NA19240	YRI	female	WUGSC	NimbleGen SeqCap_EZ_Exome v3	ILLUMINA HiSeq 2000	0,211
NA06985	CEU	female	BCM	HSGC VCRome	ILLUMINA HiSeq 2000	0,202
NA12489	CEU	female	BCM	HSGC VCRome	ILLUMINA HiSeq 2000	0,202
NA12156	CEU	female	BCM	HSGC VCRome	ILLUMINA HiSeq 2000	0,199
NA10851	CEU	male	BCM	HSGC VCRome	ILLUMINA HiSeq 2000	0,186
NA11995	CEU	female	BCM	HSGC VCRome	ILLUMINA HiSeq 2000	0,159
NA19099	YRI	female	BCM	HSGC VCRome	ILLUMINA HiSeq 2000	0,151

* BI - Broad Institute, BMC - Baylor College of Medicine, WUGSC - Washington University Genome Center

Supplementary Table S2. Validation sets for NA12878 sample

Name	Number of CNV calls	Reference	Description	Source	Processing
mccarroll2006	16	McCarroll et al. Nature Genetics, 2006	CNV discovery: HapMap (release 16) SNP genotype data without quality control filtering. Validation: FISH, Illumina BeadArray, PCR amplification and quantitative PCR	ftp://ftp.ebi.ac.uk/pub/databases/dgva/nstd20 & McCarroll & et & al & 2006/gvf/nstd20 & McCarroll & et & al & 2006.2015-11-02.GRCh37.Remapped.gvf	
conrad2006	11	Conrad et al. Nature Genetics, 2006	CNV discovery: HapMap (release 16) SNP genotype data without quality control filtering. Validation: custom CGH Array (control sample NA10851), Real-Time PCR	ftp://ftp.ebi.ac.uk/pub/databases/dgva/nstd17 & Conrad & et & al & 2006/gvf/nstd17 & Conrad & et & al & 2006.2015-11-02.GRCh37.Remapped.gvf	
redon	61	Redon et al. Nature, 2006	CNV discovery: Affymetrix GeneChip Human Mapping 500K Array Set (474,642 SNPs), Whole Genome TilePath array (26,574 clones). Control samples - NA10851,NA15510 . Validation: quantitative PCR, mass spectrometry	ftp://ftp.ebi.ac.uk/pub/databases/dgva/estd1 & Redon & et & al & 2006/gvf/estd1 & Redon & et & al & 2006.2014-04-01.GRCh37.Remapped.gvf	
wang	25	Wang et al. Genome Research, 2007	CNV discovery: Hapmap genotype data (Illumina HumanHap550 Bead Chip, PennCNV. Breakpoints by PCR and resequencing techniques	ftp://ftp.ebi.ac.uk/pub/databases/dgva/nstd64 & Wang & et & al & 2007/gvf/nstd64 & Wang & et & al & 2007.2017-10-03.GRCh37.Remapped.gvf	
pinto	9	Pinto et al. Human Molecular Genetics, 2007	CNV discovery: PopGen project data genotyped by Affymetrix 500 K SNP array set (algorithms CNAG, dChip, GEMCA). Validation: confirmed by other studies	ftp://ftp.ebi.ac.uk/pub/databases/dgva/estd55 & Pinto & et & al & 2007/gvf/estd55 & Pinto & et & al & 2007.2014-04-02.GRCh37.Remapped.gvf	

cooper	48	Cooper et al. Nature Genetics, 2008	CNV discovery: Illumina Human 1M Array (algorithm HMMseq). Validation: fosmid ESP map, oligo array-CGH	ftp://ftp.ebi.ac.uk/pub/databases/dgva/nstd14 & Cooper & et & al & 2008/gvf/nstd14 & Cooper & et & al & 2008.2015-11-02.GRCh37.Remapped.gvf	
phase1	929	The 1000 Genomes Project Consortium Nature, 2012	SV discovery: Illumina Genome Analyzer, Illumina Genome AnalyzerII, Illumina Genome AnalyzerIIx, Illumina HiSeq 2000, Roche 454 GS-FLX+, AB SOLiD(BreakDancer, CNVnator, Delly, Genome STRiP and Pinde algorithms). Genotyping: Genome STRiP. Validation: PCR, aCGH, Illumina Omni 2.5 array.	ftp://ftp.ebi.ac.uk/pub/databases/dgva/estd199 & 1000 & Genomes & Consortium & Phase & 1/gvf/estd199 & 1000 & Genomes & Consortium & Phase & 1.2013-06-27.GRCh37.Submitted.gvf	convert GVF to BED, merge overlapped CNV
mccarroll2008	1275	McCarroll et al. Nature Genetics, 2008	CNV discovery: HapMap SNP Affymetrix Genome-Wide Human SNP 6.0 Array(Birdseye). Validation: CNV set by Kidd 2008, qPCR	Supplementary Tables 2,3	
hapmap	842	International HapMap 3 Consortium, et al. Nature, 2010	CNV discovery: Affymetrix Human SNP array 6.0 and the Illumina Human1M-single beadchip (QuantiSNP, Birdseye algorithms). CGH data from Human Genome Structural Variation consortium for thresholds definition. CNV genotyping : CNVtools and two-dimensional genotyping approah	ftp://ftp.ncbi.nlm.nih.gov/hapmap/cnv & data/hm3 & cnv & submission.txt	hg18 genomic coordinates liftover to hg19, CN=2 as no CNV(0), otherwise CNV(1)
conrad2010	4586	Conrad et al. Nature, 2010	CNV discovery: 42mCGH Array (GADA),NA10851 - reference. Genotyping CNV: custom Agilent 105kCGH CNV Genotyping Array - 10 cell lines reference. Validation: CNV-typing data, prior CNV sets, qPCR, mass spectrometry, Human660W array	Supplementary Table 2	CN=2 as no CNV(0), otherwise CNV (1). Merge CNV and no-CNV regions separately, generate set of non-overlaped regions (bedtools multiinter), we assumed that regions with uncertain status are CNV

pilot	7054	The 1000 Genomes Project Consortium Nature, 2010 Mills et al. Nature, 2011	SV discovery: WGS sequencing on Illumina, 454 and Solid platforms (different mapping and CNV detection methods). Genotyping: Genome STRiP. Validation: PCR (control sample NA15510), array-CGH (Agilent 1M and 20 2.1M Nimblegen arrays), "SuperArray": data from Affymetrix 6.0 and aCGH-arrays, sequence capture (custom Nimblegen, 454 GS FLX Titanium)	ftp://ftp-trace.ncbi.nih.gov/1000genomes/ftp/pilot & data/paper & data & sets/companion & papers/mapping & structural & variation/MasterValidation.Pilot2.all.leftmost.061510.txt	Filter inversions, insertions, variation and "unvalidated" variations (VALIDATION & STATUS). "invalidated" as no CNV(0), "validated" as CNV (1). As CNV coordinates were used 'START & CI & BKPT' and 'END & CI & BKPT'. Merge CNV and no-CNV regions separately, generate set of non-overlaped regions (bedtools multiinter), we assumed that regions with uncertain status are CNV. Liftover genomic coordinates to hg19
phase3	36541	Sudmant et al. Nature,2015	SV discovery: Illumina WGS sequencing (BreakDancer, Delly, VariationHunter, CNVnator, Read-depth, Genome STRiP,Pindel, MELT, Dinumt). Genotyping: Genome STRiP. VAlidation; PCR,long-read data, SNP arrays, Agilent 1M CGH microarray (reference sample NA10851)	ftp://ftp.1000genomes.ebi.ac.uk/vol1/ftp/phase3/integrated & sv & map/ALL.wgs.mergedSV.v8.20130502.svs.genotypes.vcf.gz	work with CNV, DUP, DEL , DEL & ALU, DEL & LINE1, DEL & SVA, DEL & HERV (SVTYPE). Based on genotype data we defined CN=2 as no CNV, otherwise CNV. Merge CNV and non-CNV regions separately, generate set of non-overlaped regions (bedtools multiinter). We assumed that regions with uncertain status are CNV
lumpy	4251	Layer et al. Genome Biology, 2014	SV discovery: LUMPY predictions and the 1000 Genomes Project SV set. Validation:long-read data from from PacBio and/or Illumina Moleculo	https://static-content.springer.com/esm/art%3A10.1186%2Fgb-2014-15-6-r84/MediaObjects/13059 & 2013 & 3363 & MOESM5 & ESM.zip	filter inversions and structural variations with unknown type; convert BEDPE to BED, using outer start/end
pacbio	20957	Pendleton et al. Nature Methods 2015	SV discovery: long-read(de novo sequence assembly and read-mapping approaches) and short read data (Delly), Validation:PCR, WGS Moleculo data	ftp://ftp-trace.ncbi.nlm.nih.gov/giab/ftp/data/NA12878/NA12878 & PacBio & MtSinai/NA12878.sorted.vcf.gz	
metasv	16987		SV discovery: Platinum Genomes 2x100bps HiSeq, Illumina HiSeq (250	ftp://ftp-trace.ncbi.nlm.nih.gov/giab/ftp/technical/svclassify & Manuscript/Supplementary & Information/metasv & trio & validation/NA12878 & svs.vcf.gz	convert VCF to BED

svclassify	2676	Parikh et al. BMC Genomics, 2016	[illegible] bp), PacBio,Moleculo WGS data, Personalis deletions calls, 1000 Genomes Project pilot phase SV set. One-class classification of candidate SVs. Validation: MetaSV(Mohiyuddin et al. 2015) results, PCR validation	ftp://ftp-trace.ncbi.nlm.nih.gov/giab/ftp/technical/svclassify & Manuscript/Supplementary & Information/Personalis & 1000 & Genomes & deduplicated & deletions.bed	

Supplementary Table S3. Pairwise comparison of validation sets.

Elements on the main diagonal indicate the set size, for other elements the denominator of a fraction is the number of shared exons in the sets and the numerator is the number of exons with the same state

**Studies**	***mccarroll2006***	***conrad2006***	***phase1***	***svclassify***	***wang2007***	***cooper2008***	***pinto2007***	***hapmap2010***	***redon2006***	***mccarroll2008***	***pacbio***	***lumpymills***	***conrad2009***	***lumpy***	***metasv***	***pilot***	***phase3***
mccarroll2006	9																
conrad2006	3/3	10															
phase1	3/3	4/4	91														
svclassify	1/1	1/1	58/58	184													
wang2007	8/8	4/4	4/4	1/1	333												
cooper2008	8/8	4/4	5/5	4/4	168/168	377											
pinto2007	5/5	0/0	1/1	0/0	261/261	201/201	868										
hapmap2010	1/1	6/6	19/20	41/41	66/66	71/71	60/92	1970									
redon2006	3/3	3/3	7/7	10/10	95/95	121/121	640/640	191/309	3780								
mccarroll2008	1/1	6/6	18/19	41/43	67/87	113/150	60/167	1211/1282	204/466	3875							
pacbio	4/4	4/4	66/66	121/121	43/43	96/96	45/45	170/285	588/588	190/393	6546						
lumpymills	9/9	4/4	75/75	154/154	220/220	225/225	378/378	238/546	538/538	324/602	762/762	6697					
conrad2009	4/4	4/4	41/46	81/85	245/245	189/196	269/391	975/1228	395/761	1192/1514	541/1003	664/1235	8230				
lumpy	4/4	4/4	67/67	120/120	38/38	84/84	43/43	86/202	278/278	107/270	612/612	824/824	371/1290	18552			
metasv	9/9	10/10	69/69	174/174	221/221	275/275	413/413	334/955	914/914	539/1171	1356/1356	2871/2871	1012/2271	2194/2194	24306		
pilot	9/9	4/10	84/88	164/174	239/267	268/291	412/433	406/925	1048/1241	651/1276	1024/1638	6307/6321	1368/2431	1081/2266	3680/6198	28104	
phase3	4/4	10/10	37/52	103/124	81/98	50/118	48/167	1355/1522	261/998	2272/2450	257/1590	260/2078	3142/3847	238/3464	434/5395	3808/6176	54137

Supplementary Table S4. Intersection of algorithms calls

#ALG	Total number of exons	Combination	Number of unique exons for combination
1	69319	exomeCopy	47445
		FishingCNV	20530
		EXCAVATOR2	1096
		ExonDel	70
		CANOES	55
		ExomeDepth	50
		CODEX	17
		CNVkit	14
		PatternCNV	12
		cn.MOPS	11
		CONTRA	7
		RefCNV	5
		CLAMMS	5
		DeAnnCNV	1
		XHMM	1
2	22973	exomeCopy & FishingCNV	21314
		exomeCopy & EXCAVATOR2	1086
		EXCAVATOR2 & FishingCNV	94
		ExonDel & EXCAVATOR2	76
		exomeCopy & CANOES	72
		exomeCopy & XHMM	51
		ExomeDepth & exomeCopy	36
		exomeCopy & RefCNV	26
		exomeCopy & CNVkit	24
		CODEX & exomeCopy	21
		PatternCNV & FishingCNV	21
		ExomeDepth & EXCAVATOR2	20
		CANOES & EXCAVATOR2	16
		exomeCopy & PatternCNV	16
		CODEX & EXCAVATOR2	16
		CODEX & FishingCNV	12
		exomeCopy & CLAMMS	11
		CONTRA & FishingCNV	8
		ExomeDepth & FishingCNV	7
		cn.MOPS & EXCAVATOR2	6
		CLAMMS & FishingCNV	5
		CONTRA & exomeCopy	5
		EXCAVATOR2 & XHMM	5
		CONTRA & ExomeDepth	5

		FishingCNV & CNVkit	4
		EXCAVATOR2 & CNVkit	3
		ExomeDepth & cn.MOPS	3
		CONTRA & EXCAVATOR2	3
		CODEX & CNVkit	2
		cn.MOPS & exomeCopy	2
		CODEX & CONTRA	1
		CONTRA & PatternCNV	1
		CODEX & ExomeDepth	1
3	982	exomeCopy & EXCAVATOR2 & FishingCNV	485
		exomeCopy & CANOES & FishingCNV	82
		exomeCopy & FishingCNV & CoNIFER	62
		ExomeDepth & exomeCopy & EXCAVATOR2	57
		CODEX & EXCAVATOR2 & FishingCNV	44
		CODEX & exomeCopy & EXCAVATOR2	41
		ExomeDepth & exomeCopy & FishingCNV	21
		exomeCopy & PatternCNV & FishingCNV	21
		ExonDel & EXCAVATOR2 & CoNIFER	16
		ExomeDepth & cn.MOPS & EXCAVATOR2	14
		exomeCopy & cn.MOPS & EXCAVATOR2	12
		CODEX & ExomeDepth & EXCAVATOR2	12
		CONTRA & exomeCopy & EXCAVATOR2	11
		ExonDel & exomeCopy & FishingCNV	10
		CODEX & ExomeDepth & exomeCopy	10
		ExomeDepth & exomeCopy & RefCNV	9
		ExomeDepth & EXCAVATOR2 & FishingCNV	7
		exomeCopy & CLAMMS & FishingCNV	7
		CODEX & EXCAVATOR2 & CNVkit	6
		ExomeDepth & EXCAVATOR2 & CNVkit	6
		ExomeDepth & cn.MOPS & exomeCopy	4
		CONTRA & exomeCopy & FishingCNV	4
		exomeCopy & XHMM & CoNIFER	4
		CODEX & exomeCopy & FishingCNV	4
		ExomeDepth & PatternCNV & EXCAVATOR2	3
		CONTRA & EXCAVATOR2 & ExomeDepth	2
		ExomeDepth & exomeCopy & PatternCNV	2
		cn.MOPS & exomeCopy & FishingCNV	2
		ExomeDepth & PatternCNV & FishingCNV	2
		CONTRA & PatternCNV & ExomeDepth	2
		CODEX & ExomeDepth & ExonDel	2
		CONTRA & FishingCNV & ExomeDepth	2
		CODEX & CONTRA & FishingCNV	2
		CODEX & CONTRA & ExomeDepth	1
		CONTRA & exomeCopy & RefCNV	1

		ExomeDepth & CONTRA & CNVkit	1
		CONTRA & exomeCopy & ExomeDepth	1
		exomeCopy & XHMM & FishingCNV	1
		CODEX & CONTRA & EXCAVATOR2	1
		CODEX & ExomeDepth & FishingCNV	1
		ExomeDepth & exomeCopy & XHMM	1
		exomeCopy & RefCNV & FishingCNV	1
		exomeCopy & PatternCNV & EXCAVATOR2	1
		CONTRA & PatternCNV & CNVkit	1
		CODEX & ExomeDepth & cn.MOPS	1
		CONTRA & PatternCNV & FishingCNV	1
		CODEX & CONTRA & exomeCopy	1
4	201	CODEX & ExomeDepth & exomeCopy & EXCAVATOR2	23
		CODEX & exomeCopy & EXCAVATOR2 & FishingCNV	20
		exomeCopy & cn.MOPS & EXCAVATOR2 & ExomeDepth	16
		CODEX & ExomeDepth & EXCAVATOR2 & FishingCNV	15
		CODEX & ExomeDepth & EXCAVATOR2 & CNVkit	11
		ExomeDepth & exomeCopy & EXCAVATOR2 & FishingCNV	9
		CODEX & ExomeDepth & exomeCopy & RefCNV	6
		CODEX & FishingCNV & EXCAVATOR2 & CoNIFER	6
		ExonDel & exomeCopy & EXCAVATOR2 & FishingCNV	6
		CONTRA & exomeCopy & EXCAVATOR2 & ExomeDepth	6
		ExomeDepth & exomeCopy & CLAMMS & FishingCNV	5
		CODEX & ExomeDepth & cn.MOPS & CLAMMS	4
		ExomeDepth & exomeCopy & XHMM & FishingCNV	4
		CODEX & CONTRA & exomeCopy & ExomeDepth	4
		ExomeDepth & cn.MOPS & EXCAVATOR2 & FishingCNV	4
		CONTRA & exomeCopy & EXCAVATOR2 & FishingCNV	4
		CONTRA & cn.MOPS & exomeCopy & FishingCNV	4
		ExomeDepth & cn.MOPS & PatternCNV & exomeCopy	4
		CODEX & ExonDel & EXCAVATOR2 & CNVkit	4
		CODEX & CONTRA & EXCAVATOR2 & ExomeDepth	3
		CODEX & ExomeDepth & cn.MOPS & EXCAVATOR2	3
		ExomeDepth & cn.MOPS & PatternCNV & EXCAVATOR2	3
		CONTRA & PatternCNV & EXCAVATOR2 & ExomeDepth	3
		CODEX & ExonDel & exomeCopy & EXCAVATOR2	3
		CODEX & ExomeDepth & exomeCopy & PatternCNV	2
		CODEX & cn.MOPS & EXCAVATOR2 & FishingCNV	2
		ExomeDepth & exomeCopy & PatternCNV & EXCAVATOR2	2
		CODEX & CONTRA & EXCAVATOR2 & CNVkit	2
		ExomeDepth & exomeCopy & PatternCNV & FishingCNV	2
		CODEX & CONTRA & FishingCNV & ExomeDepth	2
		CONTRA & FishingCNV & PatternCNV & ExomeDepth	2
		ExomeDepth & exomeCopy & FishingCNV & CoNIFER	2

		CONTRA & cn.MOPS & exomeCopy & EXCAVATOR2	2
		CONTRA & exomeCopy & PatternCNV & ExomeDepth	2
		CODEX & ExomeDepth & exomeCopy & CLAMMS	1
		CODEX & CONTRA & cn.MOPS & ExomeDepth	1
		CODEX & cn.MOPS & exomeCopy & FishingCNV	1
		CODEX & CONTRA & exomeCopy & FishingCNV	1
		ExonDel & ExomeDepth & EXCAVATOR2 & CoNIFER	1
		CONTRA & exomeCopy & FishingCNV & ExomeDepth	1
		CONTRA & exomeCopy & PatternCNV & FishingCNV	1
		CODEX & CONTRA & exomeCopy & EXCAVATOR2	1
		ExomeDepth & PatternCNV & EXCAVATOR2 & CNVkit	1
		CONTRA & exomeCopy & RefCNV & ExomeDepth	1
		ExonDel & ExomeDepth & EXCAVATOR2 & CNVkit	1
5	82	CODEX & ExomeDepth & exomeCopy & EXCAVATOR2 & FishingCNV	15
		CODEX & ExomeDepth & ExonDel & EXCAVATOR2 & CNVkit	8
		CODEX & exomeCopy & cn.MOPS & EXCAVATOR2 & ExomeDepth	7
		ExomeDepth & exomeCopy & PatternCNV & EXCAVATOR2 & FishingCNV	4
		CODEX & CONTRA & FishingCNV & EXCAVATOR2 & CoNIFER	4
		CODEX & ExomeDepth & cn.MOPS & EXCAVATOR2 & CNVkit	4
		CODEX & CONTRA & exomeCopy & EXCAVATOR2 & ExomeDepth	4
		exomeCopy & cn.MOPS & RefCNV & EXCAVATOR2 & ExomeDepth	4
		CODEX & ExomeDepth & PatternCNV & EXCAVATOR2 & FishingCNV	3
		CONTRA & cn.MOPS & exomeCopy & EXCAVATOR2 & ExomeDepth	3
		exomeCopy & cn.MOPS & FishingCNV & EXCAVATOR2 & ExomeDepth	3
		ExomeDepth & exomeCopy & FishingCNV & CLAMMS & CoNIFER	3
		CODEX & CONTRA & PatternCNV & EXCAVATOR2 & ExomeDepth	2
		ExomeDepth & FishingCNV & EXCAVATOR2 & XHMM & CNVkit	2
		CODEX & CONTRA & exomeCopy & EXCAVATOR2 & FishingCNV	2
		ExomeDepth & exomeCopy & cn.MOPS & EXCAVATOR2 & CNVkit	1
		CONTRA & exomeCopy & PatternCNV & EXCAVATOR2 & ExomeDepth	1
		CONTRA & exomeCopy & FishingCNV & PatternCNV & ExomeDepth	1
		exomeCopy & cn.MOPS & FishingCNV & EXCAVATOR2 & CNVkit	1
		ExomeDepth & exomeCopy & CANOES & EXCAVATOR2 & FishingCNV	1
		CODEX & CONTRA & PatternCNV & HMZDelFinder & ExomeDepth	1
		CODEX & exomeCopy & PatternCNV & EXCAVATOR2 & FishingCNV	1
		CONTRA & cn.MOPS & FishingCNV & EXCAVATOR2 & ExomeDepth	1
		ExomeDepth & CONTRA & exomeCopy & FishingCNV & CoNIFER	1
		CODEX & CONTRA & FishingCNV & PatternCNV & ExomeDepth	1
		CODEX & CONTRA & FishingCNV & EXCAVATOR2 & ExomeDepth	1
		CONTRA & exomeCopy & PatternCNV & EXCAVATOR2 & FishingCNV	1
		CODEX & ExomeDepth & cn.MOPS & EXCAVATOR2 & FishingCNV	1
		CODEX & CONTRA & exomeCopy & CLAMMS & ExomeDepth	1
		ExonDel& CODEX & ExomeDepth & EXCAVATOR2 & CoNIFER & CNVkit	17
		CODEX & ExomeDepth & XHMM & FishingCNV & EXCAVATOR2 & exomeCopy	11

6	79	CODEX & ExomeDepth & XHMM & FishingCNV & EXCAVATOR2 & CNVkit	10
		PatternCNV & CODEX & ExomeDepth & FishingCNV & CONTRA & EXCAVATOR2	6
		CODEX & ExomeDepth & CONTRA & EXCAVATOR2 & cn.MOPS & exomeCopy	5
		CODEX & ExomeDepth & EXCAVATOR2 & exomeCopy & cn.MOPS & CNVkit	4
		CODEX & ExomeDepth & FishingCNV & EXCAVATOR2 & exomeCopy & cn.MOPS	4
		PatternCNV & CODEX & ExomeDepth & EXCAVATOR2 & exomeCopy & cn.MOPS	3
		PatternCNV & CODEX & ExomeDepth & FishingCNV & EXCAVATOR2 & exomeCopy	3
		CODEX & ExomeDepth & FishingCNV & CONTRA & EXCAVATOR2 & exomeCopy	3
		CODEX & ExomeDepth & FishingCNV & CONTRA & EXCAVATOR2 & CNVkit	2
		ExonDel & PatternCNV & CODEX & ExomeDepth & EXCAVATOR2 & CNVkit	2
		PatternCNV & CODEX & ExomeDepth & CONTRA & EXCAVATOR2 & exomeCopy	2
		PatternCNV & CODEX & ExomeDepth & CONTRA & EXCAVATOR2 & cn.MOPS	2
		PatternCNV & CODEX & ExomeDepth & EXCAVATOR2 & cn.MOPS & CNVkit	1
		PatternCNV & ExomeDepth & FishingCNV & EXCAVATOR2 & exomeCopy & CNVkit	1
		ExomeDepth & FishingCNV & CANOES & EXCAVATOR2 & exomeCopy & cn.MOPS	1
		CODEX & ExomeDepth & FishingCNV & HMZDelFinder & EXCAVATOR2 & exomeCopy	1
		ExomeDepth & FishingCNV & CONTRA & CoNIFER & exomeCopy & CLAMMS	1
7	34	CODEX & ExomeDepth & XHMM & FishingCNV & CONTRA & EXCAVATOR2 & CNVkit	6
		PatternCNV & CODEX & ExomeDepth & CONTRA & EXCAVATOR2 & cn.MOPS & CNVkit	5
		CODEX & ExomeDepth & CONTRA & EXCAVATOR2 & cn.MOPS & exomeCopy & CNVkit	3
		PatternCNV & CODEX & ExomeDepth & FishingCNV & CONTRA & EXCAVATOR2 & cn.MOPS	3
		CODEX & ExomeDepth & FishingCNV & EXCAVATOR2 & exomeCopy & cn.MOPS & CNVkit	3
		ExomeDepth & FishingCNV & CONTRA & HMZDelFinder & EXCAVATOR2 & cn.MOPS & exomeCopy	1
		CODEX & ExomeDepth & FishingCNV & CANOES & EXCAVATOR2 & exomeCopy & cn.MOPS	1
		CODEX & ExomeDepth & XHMM & FishingCNV & CONTRA & EXCAVATOR2 & exomeCopy	1
		PatternCNV & CODEX & ExomeDepth & FishingCNV & EXCAVATOR2 & exomeCopy & cn.MOPS	1
		PatternCNV & ExomeDepth & FishingCNV & CONTRA & CANOES & EXCAVATOR2 & exomeCopy	1
		PatternCNV & CODEX & ExomeDepth & FishingCNV & CONTRA & EXCAVATOR2 & exomeCopy	1
		DeAnnCNV & CODEX & ExomeDepth & RefCNV & FishingCNV & exomeCopy & CLAMMS	1
		ExonDel & PatternCNV & CODEX & ExomeDepth & EXCAVATOR2 & CoNIFER & CNVkit	1
		ExonDel & CODEX & ExomeDepth & CONTRA & EXCAVATOR2 & CoNIFER & CNVkit	1
		CODEX & ExomeDepth & FishingCNV & CONTRA & EXCAVATOR2 & cn.MOPS & exomeCopy	1
		PatternCNV & CODEX & ExomeDepth & CONTRA & EXCAVATOR2 & cn.MOPS & exomeCopy	1
		PatternCNV & ExomeDepth & FishingCNV & CONTRA & EXCAVATOR2 & cn.MOPS & exomeCopy	1
		PatternCNV & CODEX & ExomeDepth & XHMM & FishingCNV & EXCAVATOR2 & CNVkit	1
		PatternCNV & CODEX & ExomeDepth & FishingCNV & CONTRA & HMZDelFinder & exomeCopy	1
8	13	PatternCNV & CODEX & ExomeDepth & FishingCNV & CONTRA & EXCAVATOR2 & cn.MOPS & exomeCopy	3
		PatternCNV & CODEX & ExomeDepth & CONTRA & EXCAVATOR2 & cn.MOPS & exomeCopy & CNVkit	2
		PatternCNV & CODEX & ExomeDepth & FishingCNV & EXCAVATOR2 & exomeCopy & cn.MOPS & CNVkit	2
		CODEX & ExomeDepth & XHMM & FishingCNV & CONTRA & EXCAVATOR2 & cn.MOPS & exomeCopy	1
		PatternCNV & CODEX & ExomeDepth & FishingCNV & CONTRA & HMZDelFinder & EXCAVATOR2 & exomeCopy	1
		PatternCNV & CODEX & ExomeDepth & RefCNV & FishingCNV & EXCAVATOR2 & exomeCopy & cn.MOPS	1
		DeAnnCNV & CODEX & ExomeDepth & RefCNV & FishingCNV & CONTRA & exomeCopy & CLAMMS	1
		PatternCNV & CODEX & ExomeDepth & XHMM & FishingCNV & EXCAVATOR2 & exomeCopy & cn.MOPS	1

		PatternCNV & ExomeDepth & FishingCNV & CONTRA & HMZDelFinder & EXCAVATOR2 & cn.MOPS & exomeCopy	1
9	8	PatternCNV & CODEX & ExomeDepth & FishingCNV & CONTRA & EXCAVATOR2 & cn.MOPS & exomeCopy & CNVkit	4
		PatternCNV & CODEX & ExomeDepth & FishingCNV & CONTRA & HMZDelFinder & EXCAVATOR2 & cn.MOPS & exomeCopy	1
		DeAnnCNV & PatternCNV & CODEX & ExomeDepth & RefCNV & FishingCNV & CONTRA & exomeCopy & CLAMMS	1
		PatternCNV & CODEX & ExomeDepth & RefCNV & FishingCNV & CONTRA & EXCAVATOR2 & cn.MOPS & exomeCopy	1
		PatternCNV & CODEX & ExomeDepth & FishingCNV & CONTRA & HMZDelFinder & EXCAVATOR2 & CoNIFER & cn.MOPS	1
10	1	PatternCNV & CODEX & ExomeDepth & XHMM & FishingCNV & CONTRA & CANOES & EXCAVATOR2 & cn.MOPS & exomeCopy	1
11	2	PatternCNV & CODEX & ExomeDepth & XHMM & FishingCNV & CONTRA & CANOES & HMZDelFinder & EXCAVATOR2 & cn.MOPS & CNVkit	2

Supplementary Table S5. Paired comparasion of CNV calling tools.

Elements on the main diagonal indicate the number of CNV-exons predicted by tool, for other elements the number of the same predictions with other tools

Tool	CANOES	CLAMMS	cn.MOPS	CNVkit	CODEX	CoNIFER	CONTRA	DeAnnCNV	EXCAVATOR2	exomeCopy	ExomeDepth	ExonDel	FishingCNV	HMZDelFinder	PatternCNV	RefCNV	XHMM
**CANOES**	232																
**CLAMMS**	0	46															
**cn.MOPS**	5	4	185														
**CNVkit**	2	0	32	163													
**CODEX**	4	9	81	103	496												
**CoNIFER**	0	4	1	19	30	120											
**CONTRA**	4	4	50	29	98	8	192										
**DeAnnCNV**	0	3	0	0	3	0	2	4									
**EXCAVATOR2**	23	0	148	117	391	47	120	0	3496								
**exomeCopy**	159	32	113	45	239	73	103	3	1905	71228							
**ExomeDepth**	7	18	142	102	281	28	123	3	419	357	637						
**ExonDel**	0	0	0	34	38	36	1	0	136	19	33	218					
**FishingCNV**	89	24	54	38	219	80	90	3	819	22164	211	16	42979				
**HMZDelFinder**	2	0	6	2	8	1	9	0	8	6	10	0	9	10			
**PatternCNV**	4	1	44	23	64	2	61	1	81	95	101	3	100	8	178		
**RefCNV**	0	3	6	0	11	0	5	3	6	53	25	0	6	0	3	58	
**XHMM**	3	0	5	21	34	4	11	0	41	76	41	0	41	2	5	0	103

Supplementary Table S6. Number of predicted CNV-exons confirmed by other tools

Tool	Number of confirmations			
	0	*1-2*	*3-6*	*>6*
PatternCNV	12	71	73	22
CONTRA	7	54	111	20
CLAMMS	5	23	16	2
HMZDelFinder	0	0	4	6
FishingCNV	20530	22224	203	22
ExomeDepth	50	233	330	24
CODEX	17	179	277	23
CoNIFER	0	82	37	1
cn.MOPS	11	44	109	21
ExonDel	70	104	44	0
XHMM	1	62	35	5
DeAnnCNV	1	0	1	2
EXCAVATOR2	1096	2043	335	22
CANOES	55	170	4	3
CNVkit	14	47	92	10
exomeCopy	47445	23519	243	21

Supplementery Table S7. Efficiency of CNV calling tools

Tool	Precision	Recall	F1-score
CANOES	0,039	0,002	0,004
CLAMMS	0,633	0,009	0,018
cn.MOPS	0,804	0,065	0,120
CNVkit	0,679	0,044	0,082
CODEX	0,581	0,117	0,195
CoNIFER	0,825	0,025	0,048
CONTRA	0,580	0,044	0,081
DeAnnCNV	1,000	0,001	0,003
EXCAVATOR2	0,351	0,480	0,406
exomeCopy	0,052	0,646	0,097
ExomeDepth	0,585	0,154	0,244
ExonDel	0,639	0,051	0,094
FishingCNV	0,040	0,267	0,069
HMZDelFinder	0,800	0,004	0,008
PatternCNV	0,556	0,034	0,063
XHMM	0,400	0,019	0,037
